# Connect to work programme in Greater Essex: evaluation framework and study protocol

**DOI:** 10.3389/fpubh.2026.1884356

**Published:** 2026-07-31

**Authors:** Julia Gawronska, Dimitra Mouriki, Hannah Talor, Alastair Gordon, Johnathon Cuthbertson, Sarah Muckle, Lee Smith

**Affiliations:** 1 Health Determinants Research Collaboration Greater Essex, Essex County Council, Chelmsford, United Kingdom; 2 Centre for Health, Performance and Wellbeing, Anglia Ruskin University, Cambridge, United Kingdom; 3 Institute of Public Health and Wellbeing, University of Essex, Colchester, United Kingdom; 4 Essex County Council, Chelmsford, United Kingdom; 5 Faculty of Medicine, Department of Public Health, Biruni University, Istanbul, Türkiye

**Keywords:** connect to work, employment, evaluation, Greater Essex, public health

## Abstract

**Introduction:**

Employment is not merely a means of financial security. It is a key social determinant of health. The Connect to Work (CtW) programme aims to support people with disabilities, long-term health conditions, and other barriers to find, secure, and sustain meaningful employment. The CtW was launched in November 2024 as the commitment and investment in England and Wales to support the Get Britain Working Again white paper. The CtW programme in Greater Essex commenced in August 2025 and has since engaged several hundred individuals across the region. The primary aim of this study is to assess the employment-related changes, including progression into work, sustaining employment, and preventing job loss, during the programme's first year. The findings will inform subsequent years of the programme delivery and development, while contributing to the evidence base on supporting people with barriers to employment.

**Methods and analysis:**

This project will use a pre-post single arm study design and utilize a mixed-methods approach. Eligible participants are aged 18 or over and willing to engage in the CtW programme activities. The CtW programme in Greater Essex is delivered by Essex County Council in partnership with three expert providers: Essex Cares Ltd, Essex Partnership University NHS Foundation Trust, and Shaw Trust. The evaluation design includes an online survey, qualitative interviews, participant workshops, and economic evaluation. The survey is administered at baseline, with follow-up points at 3 and 12 months, and explores participants' current housing and financial situations, general health, motivation to seek employment, readiness to enter the workforce, and barriers to employment. Exit interviews with participants, programme facilitators, and wider stakeholders will capture their experiences of the programme, including barriers and facilitators encountered during implementation. Workshops with participants will map their journeys and generate better understanding of their experiences of the programme. Additionally, the economic evaluation will calculate the value of the CtW programme to the Greater Essex economy.

**Ethics and dissemination:**

The results of this study will be disseminated through peer- review publications, scientific presentations, and local dissemination activities. Ethical approval was obtained through the Anglia Ruskin University Research Ethics Committee (Reference number: ETH2526-0259).

## Introduction

Employment is not merely a means of financial security. It is a key social determinant of health, influencing individuals, families, and communities both directly and indirectly (1, 2). Conversely, unemployment is associated with adverse health outcomes, including poor mental health, reduced life satisfaction (3, 4), financial insecurity, and reduced ability to access the resources and conditions that support healthy lives (5, 6). These impacts can extend beyond individuals to affect families and communities, contributing to wider health and social inequalities (6). The quality of employment also plays a critical role as job insecurity and poor working conditions can exacerbate existing health disparities. A report by the World Health Organization (WHO) on Social Determinants of Health highlights that stable and fair employment promotes social mobility, enhances social status and supports personal development (7). According to the Office for National Statistics (ONS), the unemployment rate in the United Kingdom (UK) between May and June 2025 was 4.7%, representing 1.67 million people aged 16 to 64. The economic inactivity (people who are not working and not actively looking for work) rate for individuals aged 16 to 64 was 21.1%, accounting for 9.12 million people (8). In the 3-month period from April 1st to June 30th 2025, approximately 2.78 million people in the UK were economically inactive due to long-term sickness. This number has been rising since 2019, when there were just over two million people economically inactive for the same reason (9). Long-term sickness is the leading cause of economic inactivity in the UK, with mental health and musculoskeletal conditions being the most common underlying factors (10). Over the past 5 years, the UK workforce declined by approximately 800,000 people, mostly due to ill health and early retirement (11). These figures reflect a significant loss of opportunities in the workplace for people who are vital to the UK's future. The economic impact is substantial, with current costs estimated at £150 billion annually (10). By 2030, 600,000 more people could become economically inactive, adding additional £25 billion to working age incapacity and disability welfare spending (10). In November 2024, the UK government published the Get Britain Working white paper where the government proposed to reform employment, as well as health and skills support to reduce economic inactivity and help people transition back into meaningful work (12). The government has set a long-term ambition to achieve an 80% employment rate which would translate to over two million additional people in work based on current numbers (12). The national priority is to reduce the barriers to employment faced by disabled people and those with ill-health. By removing barriers for these groups, and supporting their transition into meaningful employment, the government will be able to reduce the adverse impact economic inactivity has on the affected people, the economy and public finances. Previous evaluations of employment interventions suggest that tailored approaches addressing both employment barriers and wider health and social needs can improve employment-related outcomes. Individual Placement and Support (IPS), an evidence-based supported employment model, has demonstrated effectiveness in supporting people with mental health conditions into competitive employment (13, 14). Large-scale UK employment programmes, including the Work Programme, Work and Health Programme, and Restart Scheme, have highlighted the importance of personalized support, employer engagement, and addressing individual barriers to employment, with evaluations reporting impacts on employment outcomes and wider factors such as confidence and motivation (15–17). However, existing evidence remains mixed due to differences in intervention models, target populations, and outcome measures, and there is limited evidence on the longer-term health, social, and economic impacts of integrated employment programmes. Further evaluation of approaches such as the Connect to Work (CtW) programme is therefore needed to understand their effectiveness and wider value.

As part of the Get Britain Working white paper, the government introduced the CtW programme in England and Wales. This new initiative from the Department for Work and Pensions (DWP) is designed to support disabled people, individuals with health conditions, and those facing complex barriers to employment in securing meaningful work. This work programme will take a collaborative, locally led approach to tackle hidden unemployment by integrating local employment as well as health and skills support services (18). Through addressing the barriers faced by certain vulnerable groups, the programme aims to promote long-term market participation for economically inactive individuals and develop a more inclusive workforce.

Greater Essex covers Essex County Council (ECC), Southend-on-Sea City Council, and Thurrock Council, with a population of over 1.8 million (19). The geography of Greater Essex is diverse with varied landscapes, made up of the urban cities, coastal resorts, busy ports and rural areas (20). The region has significant opportunities in sectors such as advanced manufacturing, green energy, or digi-tech, but there are many barriers to unlocking the area's potential. Pressure on local transport infrastructure can mean that people find it harder to access jobs, healthcare services and other opportunities. There is also a shortfall in local jobs, meaning that there are fewer high-value jobs per working-age person compared to other areas across the East and Southeast regions (21). The Greater Essex workforce tends to be less well qualified, less highly skilled compared to workforce in other areas across the East and Southeast England. This is also reflected in earnings where residents who work in Greater Essex earn less compared to those who commute to outside areas. This shortage of jobs drives inequality and reduces opportunities. The people who are the most affected are those who live in the most isolated coastal communities and who cannot travel. This also includes vulnerable groups (21). In Greater Essex, there is a significant gap in educational attainment, reaching 30% between the most and least deprived areas in the county. In Greater Essex, residents can expect to live around 63 years in good health. However, substantial inequalities exist between communities, with a gap of approximately 10 years in healthy life expectancy between the most and least advantaged areas, such as Tendring and Uttlesford (22). These disparities highlight that, despite Greater Essex's strong growth and opportunities, inequalities continue to contribute to unemployment and economic inactivity among its residents. The unemployment rate in Greater Essex was 3.3% in 2024 (21) compared to 4.8% in England in 2025 (23). To match employment levels seen in neighboring counties, the Greater Essex would need to create over 100,000 additional jobs (21). Beyond these figures are a number of people experiencing “hidden unemployment.” This includes those who are not actively looking for work but who are willing and able to work and therefore are not counted as part of the official unemployment statistics. Hidden unemployment is particularly relevant among marginalized communities, vulnerable groups, including people with disabilities, care leavers, caregivers, and those with mental health conditions. Many of these experiences prolong periods of economic inactivity, either due to lack of available job opportunities or the structural challenges associated with workforce reintegration (24).

Returning to work is a complex process, influenced by health, psychosocial and workplace factors. Individuals who have been economically inactive due to disability or long-term illness, face barriers such as their health problems, systemic discrimination, and lack of suitable workplace adjustments to ensure smooth transition and retention (25). However, targeted activities can lead to meaningful changes in employment outcomes (15–17). To support the design and evaluation of the CtW programme, we developed a programme-specific Theory of Change (ToC) (26). This framework is widely adopted across a range of policy areas to guide programme design and evaluation. It describes how a sequence of steps can lead to meaningful outcomes. In the context of the CtW programme, individuals will receive ongoing personalized support and job-matching services to help improve their job readiness and secure meaningful and sustainable employment. Individuals will also receive personalized support ranging from coaching to sessions with employment specialists, aimed at helping them gain confidence in their ability to carry out work-related tasks. The CtW programme's focus on personalized support reflects an understanding that employment readiness is not only shaped by participants skills, but it also depends on their emotional resilience, self-belief, and the availability of inclusive employment. By embedding the ToC into the evaluation process, we aim to assess whether the CtW programme effectively addresses external barriers to employment and enhances participants' capacity to engage and grow.

## Settings

ECC will act as the Accountable Body for the CtW programme leading the design and delivery for Greater Essex (Essex, Thurrock and Southend). ECC mandates the IPS and Supported Employment Quality Framework (SEQF) models. These approaches focus on providing individualized personal support that is tailored to everyone's specific needs and delivered end to end by Employment Specialists. ECC commissioned three expert providers: Essex Cares Ltd (ECL), Essex Partnership University NHS Foundation Trust (EPUT) and Shaw Trust to support programme delivery. ECL provides SEQF Inclusive Employment Programme for learning disabilities and autism into Southend and Thurrock from August 2025 and extend eligibility criteria to non-diagnosed and 16+ across Greater Essex. EPUT provides IPS for mild to moderate mental health (and other long term health conditions) into Southend and Thurrock from August 2025. Shaw Trust provides support for physical and sensory disabilities and for priority groups including Veterans, refugees, offenders, unpaid carers and care leavers with barriers to employment from January 2026. We hypothesize that involving different organizations to support distinct vulnerable groups creates opportunities for individuals to choose and secure the right job, thereby enhancing job retention.

## Aims

The primary aim of this evaluation is to assess how effectively the CtW programme supports people with disabilities, long-term health conditions, or complex barriers to enter, sustain, or retain meaningful employment in Greater Essex.

Objectives of this evaluation include:

- Barriers to employment: to identify the employment barriers faced by people with disabilities, long-term health conditions, and other vulnerable groups in Greater Essex.
- Role of the CtW: to examine how the CtW programme supports participants throughout their employment journey, including engagement over a minimum of 12 weeks.
- Short-terms impacts: to assess the short-term impacts on employment outcomes (progression into work of 16+h per week, sustained employment for at least 24 weeks, and prevention of job loss), and changes in mental health, life satisfaction, quality of life, and overall wellbeing.
- Economic value: to estimate the economic value of the CtW programme to the Greater Essex economy.
- Influencing factors: to identify the factors that positively or negatively influence participants' reintegration into employment across different demographic and health related groups.

### Primary outcomes

The proportion of unemployed participants who enter and sustain meaningful employment within 12 months of enrolment in the CtW programme, and the proportion of employed participants who retain their employment within 4 months of enrolment in the CtW programme.

A precise effect size cannot be specified *a priori*. The programme is expected to increase the proportion of participants entering sustained employment within 12 months. The magnitude of this effect will be estimated through the evaluation.

## Methods and analyses

The evaluation of the CtW programme is designed as a mixed-methods study. It includes and online survey for participants with two follow-up points, interviews with participants, programme facilitators, and wider stakeholders. In addition, we will conduct workshops with participants to map their experience and gather deeper insights.

### Online survey

Participants will be invited to complete an online survey at baseline, and again at 3-month and 12-month follow-up points (Supplementary File 1). Each participant will receive a digital information sheet and consent form, which must be signed prior to taking part. Period of involvement may vary, and each participant will follow an individual timeline for data collection and follow-up.

### Qualitative interviews

We aim to conduct semi-structured interviews with participants, programme staff, and wider stakeholders involved in the CtW programme.

Interviews with participants will take place upon their exit from the programme, while interviews with staff and stakeholders will be conducted at the end of the first year of implementation in Greater Essex. Preliminary analysis of the interview data with participants will inform revisions to the interview guides, where necessary. Approximately 25 to 30 one-to-one semi-structured interviews will be undertaken with participants when they exit the programme.

An additional 20 semi-structured interviews with programme staff and wider stakeholders will be conducted. Ten with programme staff and ten with stakeholders. Their perspectives are essential for understanding the CtW programme's relationships and its perceived impact.

The proposed number of interviews is appropriate for a qualitative evaluation of this scale, as thematic saturation is commonly reached within 20 to 30+ in-depth interviews (27). Purposive sampling will ensure representation across disability types, health conditions, and complex barriers, supporting the inclusion of marginalized groups. We will expand the sample, if early analysis indicates that additional interviews are needed.

### Experience mapping

We will conduct two to four workshops with participants to explore their experiences of the CtW programme. This approach will enable participants to collectively reflect on the process and outcomes of their engagement, highlighting how specific experiences such as staff interactions, appointments, starting a new job, or participating in training shape their overall journey.

### Economic evaluation

Our aim is to undertake an exploratory assessment of the potential economic implications of the CtW programme for the Greater Essex economy. As this evaluation does not include a control group, it will not be possible to attribute observed changes directly to the programme. Instead, the economic analysis will use observed participant outcomes alongside administrative data to estimate the potential economic value associated with changes in employment, productivity, welfare benefit use, and healthcare costs.

## Inclusion criteria

Individuals 16 years and over will be eligible to participate in the CtW programme if they have been referred and have agreed to take part. However, for the evaluation, only individuals aged 18 years and over will be included as the evaluation focuses on adult participants and involves the collection of sensitive information relating to health, wellbeing, and employment circumstances. The only exclusion criterion applies to those who were offered the CtW programme but did not engage with any of the support provided, such as attending the initial meeting or developing a support plan. The target population for this programme is adults from diverse backgrounds who may face barriers to employment. This includes individuals with mild to moderate mental health conditions, long-term health conditions (e.g., musculoskeletal disorders), learning disabilities and autism, physical and sensory disabilities, as well as veterans, (ex) offenders, unpaid carers, and care leavers.

## Recruitment

Recruitment of participants will be carried out through multiple channels. Leaflets will be distributed directly to individuals participating in the CtW programme. Posters will be displayed in organizations involved in delivering the programme to increase visibility. Programme staff will actively inform and encourage eligible participants to take part in the study during their regular interactions. Additionally, we will offer a £25 gift voucher for participation in the interview or workshop, and participants completing the online survey will be eligible to enter a draw for a £100 gift card. As the evaluation includes only individuals who have engaged with the CtW programme, there is potential for recruitment bias. Individuals who were offered the programme but did not engage with any support are excluded and may differ from those who participated. Consequently, the findings will reflect the experiences of programme participants rather than all individuals offered CtW support. To minimize selection bias among eligible participants, all individuals who engage with the programme will be invited to participate in the evaluation through our recruitment process.

It is expected that 2,600 participants will start the programme in the first year of the delivery. As CtW is a voluntary programme, recruitment depends on the number of eligible participants who choose to take part. Consequently, the sample size will be determined by programme uptake during the evaluation period, rather than by a formal power calculation.

## Procedures

The data collection process will take place over a 12-month period. A link to the online survey will be sent to participants at baseline, and again at the 3-month and 12-month follow-up points. Programme staff will support and encourage survey completion during employment sessions. A trained researcher will conduct qualitative interviews with participants, programme staff, and wider stakeholders at a date and time convenient for each individual. Interviews will last approximately 40 min. Participants will be invited to take part in one of the workshops, each involving approximately five to six individuals. The workshops are expected to last between 1 and 2 h, and participants will be free to leave at any time. All workshops will be held at a location convenient for all attendees. To ensure our study is inclusive and accessible to all participants, we will provide access to translators if needed.

## Measurement and instruments

### Survey

All participants in the study will be asked to complete an online survey, which will take approximately 15 min. The survey includes questions on standard demographics, current housing situation, housing instability (28), and financial distress (29). Additional metrics will be assessed, including general health (30), personal wellbeing (31), social connectedness and inclusion (32, 33), level of mastery (34), motivation to seek employment (35), readiness to return to work (36), barriers to employment (37), confidence in employment (38), and workplace satisfaction (39). The general health will be assessed using the SF-12 Items Short Form Survey that assesses health-related quality of life across eight domains (30). The survey demonstrates good internal reliability and validity and is a suitable tool for assessing health-related quality of life in vulnerable populations, including those with mental illness, low income, or unstable housing (40, 41). Social connectedness and inclusion will be measured using the UCLA 20-Item Loneliness Scale (32) and the Brief Sense of Community Scale (33). Both scales have demonstrated strong reliability and construct validity across diverse populations (42, 43).

Participant's personal mastery will be assed using the Pearlin Mastery Scale, which is a reliable and valid tool for assessing the extent to which individuals believe they are in control of their own lives (34, 44). Motivation to seek employment will be measured using the Job Search Self-Efficacy Scale (35). This scale is a psychometrically sound tool that refers to the belief that an individual can successfully perform job search and obtain employment (35, 45). The Readiness for Return-to-Work Scale will be used to assess participants' psychological preparedness to resume employment following injury, illness, or an extended absence (36). The scale comprises 22 items and has demonstrated sound psychometric properties, supporting its relevance and reliability in work disability research (36). Participants will also be asked to complete the Perceived Employment Barrier Scale to identify the challenges and obstacles they encounter when seeking employment (37). This instrument has demonstrated strong reliability and validity, making it a robust measure for assessing employment barriers among low-income jobseekers (37). We will explore participants confidence in employment using the Occupational Self-Efficacy Scale (short version). This tool measures the confidence an individual has in their ability to cope with difficult tasks or problems at work (38). For participants who secure supported employment through the CtW programme, we aim to explore their workplace satisfaction. This will be assessed using the Short Index of Job Satisfaction Scale (39). We anticipate that over the 3- and 12-month follow-up periods, the programme is likely to influence outcomes such as motivation to seek employment, readiness to return to work, confidence in employment, personal wellbeing, and social connectedness and inclusion. It may also partially improve barriers to employment, general health, and workplace satisfaction among participants who are employed but at risk of losing their job. More structural outcomes, such as current housing situation and housing instability, are included to characterize participants' socioeconomic circumstances rather than because they are expected to change directly as a result of the intervention.

### Qualitative interviews and workshops

Qualitative interviews with participants will focus on exploring their experiences, challenges, and perceived progress in securing employment and developing relevant skills. The interviews will also examine employment outcomes, long-term and sustainable work, perceived barriers and facilitators, and broader changes in participants' lives. An interview guide with open-ended questions will allow the researcher to ask follow-up questions for further clarification.

Qualitative interviews with programme staff will be designed around the areas of concern consistent with the CtW programme's outcomes, barriers and facilitators. This will create space for programme staff to reflect on their experiences from day-to-day work and delivery of the CtW programme. Staff will be encouraged to explore topics of importance to them.

Qualitative interviews with stakeholders will explore key areas such as engagement, the relevance of the CtW programme, perceived barriers and enablers, and shifts in perception over time. Stakeholders' insights are essential for understanding external relationships and the broader impact of the CtW programme.

Participant workshops will explore experiences of the CtW programme through visual mapping of their journeys. This process will help identify key moments, perceived barriers and facilitators, and the overall impact of the service on their lives. All workshops will be guided by a topic guide to ensure consistency while allowing flexibility for participants to share their individual perspectives.

We developed the topic guides for participant interviews, staff and stakeholder interviews, and workshops by drawing on relevant previous research, the programme's ToC, and the evaluation objectives. We also discussed the key areas of interest with stakeholders to ensure that the guides captured topics that were relevant and meaningful to the programme context. The guides were then refined to align with the research aims and objectives, while ensuring that questions complemented information already collected during referral process and via online survey. Each guide uses open-ended questions, simple language, and flexible probing to allow facilitators to explore experiences in depth while maintaining participant-centered approach. Interview and workshop guides are included in Supplementary Files 1–4.

### Economic evaluation

The economic evaluation will draw on programme administrative data, participant survey data, programme cost data, qualitative interview and workshop data. Administrative data collected during referral and programme delivery will provide information on participant characteristics, engagement with the programme, and employment outcomes. Participant surveys will capture demographics, housing stability, financial distress, general health, wellbeing, social connectedness, sense of mastery, motivation to seek work, readiness to return to work, employment barriers, confidence in employment, and where relevant workplace satisfaction. Programme financial records will be used to estimate delivery costs and potential economic implications. Qualitative interview data will provide contextual insight into participants' experiences and perceived impacts, supporting interpretation of the economic findings.

## Analysis

### Quantitative analysis

Raw data will be exported from the survey software (Jisc) into SPSS software (Version 27.0). Demographic characteristics will be summarized using descriptive statistics. Categorical variables will be compared across groups using χ^2^ tests or Fisher's exact test where cell counts are small. Continuous demographic variables will be analyzed using independent *t*-tests or ANOVA when normally distributed, and Wilcoxon rank-sum or Kruskal–Wallis tests when data are non-normally distributed. To analyse change over time in the online survey (baseline and two follow-up points), continuous outcomes will be assessed using paired tests for two time points (paired *t*-test or Wilcoxon signed-rank for non-normally distributed data). For outcomes measured across all three time points, repeated-measures ANOVA will be used, with appropriate corrections applied where required. For categorical paired data, change between two time points will be analyzed using McNemar's test, and across three time points using Cochran's *Q* test. Results will be reported with 95% confidence intervals and a significance level of 5%.

### Qualitative interviews

Each interview will be recorded and transcribed verbatim using the automated functions in Microsoft Teams. The transcripts will then be reviewed, anonymised and cleaned to eliminate errors. Interview data will be imported into qualitative data management software (NVivo 12, released in 2017). Using thematic analysis that combines both inductive and deductive approaches (46), we will explore the experiences of participants, programme staff, and stakeholders involved in the CtW programme.

### Workshops

Each workshop will be recorded using a mobile phone dictaphone and transcribed verbatim. Transcripts will then be reviewed, anonymised, and cleaned to correct any errors. Thematic analysis will be applied using an inductive approach to code each workshop transcript (47).

### Economic evaluation

An economic specialist will be commissioned to support the design and delivery of the economic evaluation. The analysis will adopt a transparent approach, with all assumptions reported and tested through sensitivity analyses to examine the robustness of the findings. Where appropriate, a social return on investment (SROI) framework will also be considered to capture the broader social and economic value associated with the programme. The economic findings will therefore be interpreted as exploratory estimates of the potential economic contribution of the CtW programme, intended to inform future service development and commissioning decisions rather than provide definitive evidence of economic impact.

## Limitations

This study will only enroll participants who consent to participate in the CtW programme. One potential limitation is a high drop-out rate, which tends to be more common among vulnerable populations compared to other groups. However, the proposed study offers a perspective that suggests greater receptiveness, potentially driven by the perceived meaningfulness of employment and its associated benefits. Furthermore, the trust built between programme staff and participants is likely to help reduce drop-out rates. Another limitation may be participants' difficulty in completing the online survey, due to lower level of digital literacy and limited internet access. To address this, programme staff will provide support in completing the survey and facilitate access to the necessary technology. Another limitation is the potential for recall bias among participants who may struggle to accurately remember past experiences or health conditions due to extended periods of unemployment. This is closely linked to reporting bias, where participants might selectively disclose or distort information because of feelings of shame or stigma associated with being economically inactive. To mitigate this, we will focus on building trusting relationships with participants and fostering a non-judgmental environment to encourage honesty and accuracy in their responses. Our use of a data triangulation approach (online surveys, interviews, and workshops) will improve consistency and strengthen the reliability of the data. A further limitation relates to the overall study design. As this study does not include a comparison group, any changes observed in participants' employment status, health, or wellbeing cannot be attributed solely to the CtW programme. Improvements may also reflect external factors, such as changes in personal circumstances, labor market conditions, or other support services. Consequently, the quantitative findings will be interpreted as associations rather than evidence of causality and will be considered alongside the qualitative data to provide a more comprehensive understanding of participants' experiences and the perceived contribution of the programme. In addition, the findings may have limited transferability beyond the study setting. Participants are recruited for a CtW programme and consist of individuals who have consented to participate, which may not be representative of all economically inactive individuals or similar employment support programmes in other settings. Furthermore, the evaluation does not capture the experiences of individuals who were offered CtW support but did not engage with the programme. These individuals may differ from participants in terms of their needs, barriers, or circumstances, which may influence access to employment support and contribute to inequalities in programme reach. Therefore, the findings will primarily reflect the experiences and outcomes of individuals who engage with CtW programme rather than all individuals eligible for the programme. An additional limitation is that no formal Patient and Public Involvement (PPI) was undertaken during the development of this study. Although the design was informed by discussions with CtW programme staff and stakeholders, individuals eligible for the programme were not involved. As a result, the study may not fully reflect participants' priorities or perspectives.

## Patient and public involvement

No formal PPI was undertaken during the development of this evaluation. The research design was informed through discussions with CtW programme staff and key stakeholders, who provided insights into the feasibility of the proposed methods, participant recruitment, and practical aspects of data collection. Their input helped ensure that the study procedures were appropriate for delivery within the programme.

Although individuals eligible for the CtW programme were not involved in the design of this study, their feedback will help inform the interpretation of findings and identify recommendations for the subsequent years of the programme. The absence of formal PPI during the study design is acknowledged as a limitation.
