# Supplementary material for: Connect to work programme in Greater Essex: evaluation framework and study protocol
Source: Front Public Health. 2026 Jul 31;14:1884356. doi: 10.3389/fpubh.2026.1884356 (PMC13473255; doi:10.3389/fpubh.2026.1884356)
Supplement: Supplementary file 1 [file Table_1.docx]

**Interview guidelines:** Connect to Work programme

Conducted with participants who participated in the Connect to Work programme.

This interview guide is for people who participated in the Connect to Work programme. Please note that the interviews will be semi-structured. The questions and flow will vary depending on the experiences reported by participants.

**USER** **interview guide**

**Introduction**

- Thank the participant for participation in the study.
- Brief description of the interview process and approximate duration.
- Explain the participant consent form, that the conservation will be audio recorded and that the interview will be anonymized.
- Participants should read and sign the consent form before the interview starts.
- Ask if there are any questions.

**Background**

1. Can you tell me a bit about yourself and your situation before joining the CtW programme? *[Prompts: What were you doing at the time-were you working, unemployed, or looking for work?]*
2. How would you describe your experience with employment have been like up to this point? *[Prompts: What types of work have you had in the past? What was positive or negative about these experiences?]*
3. Have you taken part in any other employment support programmes before CtW? *[Prompts: Which programmes-what type of support? And how was your experience of those like?]*

**Participating in the CtW programme**

1. Can you tell me, how did you first hear about the CtW programme *[Prompts: Were you referred by someone? Self-referred? When did you joined and completed the programme?]*
2. What made you decide to join the CtW programme? *[Prompts: What were you hoping it would help you with?]*
3. What were your expectations when starting the programme? *[Prompts: What did you hope to get out of it? Did you have any specific goals in mind- such as finding work, developing skills, building confidence?]*

**Barriers to employment**

1. Can you tell me about any work you had recently?
2. Have you faced any challenges or barriers in finding or staying in work? [*Prompts: Can you give an example? What were those that made it harder for you?]*
3. How would you describe the quality of your previous employment/work? *[Prompts: how well did it suit your needs? Interests, goals?]*

**Experience of the programme**

1. How would you describe your experience of taking part in the CtW programme?
2. What aspects of the CtW programme, if any, were most helpful for you? *[Prompts: if any, why? Can you please provide an example?]*
3. What aspects of the CtW programme, if any, were least helpful for you? *[Prompts: why? Can you please provide an example? Anything that felt less relevant?]*
4. In what ways, if any, has the CtW programme influenced your thinking or decisions about work in recent months? *[Prompts: Did affect the type of work you are looking for? Your goals or confidence?]*
5. Do you feel you got the support and guidance you needed from the staff?

**Experience of employment**

1. Have you worked since completing the programme, if so, what has that experience been like? *[Prompts: What kind of work was it? How is it related with your longer-term goals you might have for the future?]*
2. Do you feel more prepared for work now than before you joined the CtW? *[Prompts: in what ways? Or if not, why?]*
3. When you think about what meaningful work looks like for you, what comes to mind? *[Prompts: Are there any specific elements/factors that makes a good job for you? E.g. purpose, stability, flexibility]*

**Impact of the programme**

1. Reflecting on your experience with the CtW programme, how would you say it has shaped your personal and professional journey? *[Prompts: Have your goals/priorities changed in any way? Has it affected or been any changes in your day-today life? Or general wellbeing?]*
2. Since being part of the programme, have there been any changes in your life outside/beyond of work? If yes, in what ways? *[Prompts: can please provide an example? Do you associate these changes with takin part in the programme? Think about your daily routines, relationships, housing? What if anything has changed?]*

**Final reflections and suggestions**

1. Were there any areas that you felt that programme did not meet your expectations or needs?
2. How do you think the CtW programme could be improved for others in the future?

**End of the interview**

1. Is there anything else you would like to share or anything you thought that we haven’t already talked about today?
2. Do you have any questions for me?

Thank you very much for taking part.
