# Supplementary material for: Connect to work programme in Greater Essex: evaluation framework and study protocol
Source: Front Public Health. 2026 Jul 31;14:1884356. doi: 10.3389/fpubh.2026.1884356 (PMC13473255; doi:10.3389/fpubh.2026.1884356)
Supplement: Supplementary file 2 [file Table_2.docx]

**Interview guidelines:** Connect to Work programme

Conducted with practitioners who delivered and facilitated the Connect to Work programme.

This interview guide is for practitioners who delivered and facilitated the Connect to Work programme. Please note that the interviews will be semi-structured. The questions and flow will vary depending on the experiences reported by practitioners.

**USER** **interview guide**

**Introduction**

- Thank the participant for participation in the study.
- Brief description of the interview process and approximate duration.
- Explain the participant consent form, that the conservation will be audio recorded and that the interview will be anonymised.
- Participants should read and sign the consent form before the interview starts.
- Ask if there are any questions.

**Background**

1. Have you held roles similar to your current position before working with the CtW programme? *[Prompt: if yes, in what role? And how did it differ from the CtW programme?]*
2. Can you tell me about your role within the CtW programme? *[Prompt: what does your day-to-day work involve? Have your responsibilities changed over time? What were your specific responsibilities in the coordination and delivery of the programme?]*

**CtW programme**

1. In your experience, what are the main reasons participants decide to participate in the CtW programme? [*Prompts: are any circumstances, needs or motivations that come up often?]*
2. In your experience, what are the common reasons why participants drop out or decide stop engaging with the programme? [*Prompts: Can you provide examples of common barriers to participation or reasons for withdrawing? Can you think of any instances where participants’ expectations differed from what the CtW could provide/offer?]*
3. Based on your experience, which aspects or components of the programme have a positive impact on participants? *[Prompts: Are there any specific activities, training, or approaches that considered particularly effective?]*
4. Based on your experience, are there any elements of the programme that have been less effective or have not worked as intended?
5. What kind of challenges or difficulties have participants encountered before and while taking part in the programme? *[Prompts: were any groups / participants that had lower participation and faced greater challenges in engaging or benefiting from the programme? If so, why? How the programme support participants in overcoming those challenges?*

**Delivering the programme**

1. From your perspective, what have been the main challenges in delivering effectively the programme? *[Prompts: can you reflect on any tensions?]*
2. What factors or conditions have facilitated successful delivery of the programme? *[Prompts: reflect on working the stakeholders?]*

**Programme process**

1. Can you describe how decisions are made regarding the referrals of participants in the CtW programme? *[Prompts: what factors influence the decisions of who is accepted or declined?]*
2. Have there been changes over time in how referrals into the CtW programme are managed? *[Prompts: if so, what factors contributed to those changes?]*
3. Based on your experience, is there anything you would recommend improving about the referral and acceptance processes in CtW? *[Prompts: Please reflect on any changes that might make the process more effective]*
4. To what extent has the CtW programme successfully reached those it aims to support? *[Prompt: has the programme design reflected participant’s actual needs? Were there any groups of participants who found harder to engage? Can you please provide an example]*

**Impact and outcomes**

1. Reflecting on your experience, what changes have participants experienced as a result of engaging with the CtW programme, both related to employment and in other areas of life?
2. To what extent did the programme lead to participants finding or retaining work, training, or other positive outcomes after completion? *[Prompts: Are there any follow up mechanisms to track participants’ progress?]*
3. Do you think any changes are likely to be sustained after exiting the programme? What are the factors that can support long-term changes /impact? What follow-up/support will be in place?
4. To what extent, has your work on the CtW programme influenced the way you think about supporting vulnerable groups into employment and helping them sustain work? *[Prompts: has your understanding of the challenges faced by specific groups changed because of your involvement in the CtW? Are there any -new or different- strategies or perspectives you consider important when supporting vulnerable groups? Any changes in your practice? How well the programme addresses diverse backgrounds, identities and needs of participants?]*

**Final reflections and continuation of the programme**

1. How would you describe the overall quality of delivery of CtW programme? *[Prompt: You can reflect on the main successful elements and less successful across the programme?]*
2. Based on your experience, are there any changes that you are considering with regard to CtW programme delivery/implementation *[Prompt: Can you please provide some examples?]*

**End of the interview**

1. Is there anything else you would like to share or anything you thought that we haven’t already talked about today?
2. Do you have any questions for me?

Thank you very much for taking part.
