# Supplementary material for: Connect to work programme in Greater Essex: evaluation framework and study protocol
Source: Front Public Health. 2026 Jul 31;14:1884356. doi: 10.3389/fpubh.2026.1884356 (PMC13473255; doi:10.3389/fpubh.2026.1884356)
Supplement: Supplementary file 3 [file Table_3.docx]

**Interview guidelines:** Connect to Work programme

Conducted with stakeholders who delivered and facilitated the Connect to Work programme.

This interview guide is for stakeholders who engaged with the Connect to Work programme. Please note that the interviews will be semi-structured. The questions and flow will vary depending on the experiences reported by stakeholders.

**USER** **interview guide**

**Introduction**

- Thank the participant for participation in the study.
- Brief description of the interview process and approximate duration.
- Explain the participant consent form, that the conservation will be audio recorded and that the interview will be anonymised.
- Participants should read and sign the consent form before the interview starts.
- Ask if there are any questions.

**Context and background**

1. Can you describe me about your current role and your involvement in the CtW programme? *[Prompt: how long have you been involved? In what capacity or responsibilities? What motivated your involvement in the CtW?]*

**Understanding the programme**

1. In your own words, what do you see as the main purpose/aim of the CtW programme? *[Prompt: what did you think is trying to achieve when you first involved? Have those impression changed? How well clearly/well do you think this is communicated or understood across different stakeholders?]*
2. To what extent, and if so, in what ways, has your understanding or view about the programme has changed over time? *[Prompts: If so, why? what influenced those changes? What do you see differently now compared with when you started?]*

**Experience of the programme**

1. From your perspective, what aspects of the CtW programme have worked well? *[Prompts: Can you provide examples of specific approaches, activities, or ways of working? What do you think made them effective?]*
2. From your perspective, what aspects of the CtW programme haven’t worked as well as you had hoped/expected? *[Prompts: Can you reflect on issues, concerns, processes? What barriers have you experienced in your involvement with the programme? Wha tdo you think contributed to those challenges?]*
3. How does your experience with CtW compared with other programmes you have been involved in? *[Prompts: Can you please provide an example?*

**Connecting and supporting participants**

1. How would you describe your experience of connecting with participants or candidates through CtW programme? *[Prompts: What facilitated that connection? What barriers or gaps did you experience?]*
2. In what ways has the programme influenced your ability to recruit, train, or support people from a range of backgrounds or with varying support needs? *[****Prompts FOR EMPLOYERS****: In what ways, if any, has CtW shaped your recruitment practices? How would you describe the readiness of participants you met? What would your suggestions in relation to relevant training or skills development? What do you think would be most useful in preparation?] [****Prompts FOR TRAINING PROVIDERS:*** *To what extent, did participants have different support needs? How did you address them?]*
3. How would you describe participants engagement, retention and transitions after starting work or training? *[****FOR EMPLOYERS:*** *What aspects/factors seemed to affect whether participants stayed in the role? In your experience, what seemed to support success? Can you provide any examples?] [****FOR TRAINING PROVIDERS:*** *How did participants engage with training that you provided? What helped engaged or made them drop out?]*
4. In what ways, if any, has your knowledge and/or confidence in hiring, training, and supporting people from the groups involved in the CtW changed? [*Prompts: Can you please share an example that shows how your approach has involved?]*
5. How, if at all, has the programme influenced how you support people with different employment needs or barriers? *[Prompts: Reflect on if you have changed your engagement strategies, support systems, or expectations? What’s different now in your practice?]*

**Perception of impact**

1. Based on your experience, what kind of impact do you think the CtW programme has had on participants? *[Prompts: Reflect on job retention, employment skills, wellbeing, motivation]*
2. In what ways has the programme influenced how you approach supporting people who face barriers to employment or training? *[Prompts: Have you changed your approach, strategies or assumptions? Can pleas provide an example, where you did something differently because of your involvement in the CtW?]*
3. Have your views or understanding of the challenges or barriers faced by participants changed throughout your involvement in the programme? *[Prompts: What have you learned about participants’ needs, lived experiences, motivations? Were any barriers you had not previously considered]*

**Final reflections**

1. If you can suggest any changes to the CtW programme, what would they be, and why? *[Prompts: Reflect on any aspects of the programme:such as support mechanisms, participation, training content, stakeholder collaboration? What practical or strategic improvements would strength the programme?]*
2. Has CtW programme highlighted any wider issues within the employment or training system that potentially affected you work?

**End of the interview**

1. Is there anything else you would like to share or anything you thought that we haven’t already talked about today?
2. Do you have any questions for me?

Thank you very much for taking part.
