# Supplementary material for: Connect to work programme in Greater Essex: evaluation framework and study protocol
Source: Front Public Health. 2026 Jul 31;14:1884356. doi: 10.3389/fpubh.2026.1884356 (PMC13473255; doi:10.3389/fpubh.2026.1884356)
Supplement: Supplementary file 4 [file Table_4.docx]

**Workshop guidelines:** Connect to Work programme

The workshops aim to understand how CtW service users experience the programme. The goal is to capture both individual nuances and collective patterns of participants’ journeys.

**Workshop guide**

**Introduction**

- Thank the participant for participation in the workshop.
- Brief description of the workshop process and approximate duration.
- Explain the participant consent form, that the workshop will be audio recorded and anonymised.
- Participants should read and sign the consent form before the workshop starts.
- Ask if there are any questions.

**Experience mapping activity (35-50 mins)**

**Paper-based maps**: Large sheets with a clear journey timeline (start → middle → end), where participants place stickers, draw, or write short words.

**Start point** - Let’s think about when you first joined Connect to Work. What happened? How did you feel?

**Key moments** - What are some important things you remember? Training? Meeting staff? Interviews?

**Barriers & support** - Were there any times it felt difficult? What helped you?

**Outcomes** - What changed for you after being part of the programme?

**Reflection** - Looking back at the whole journey, how do you feel now?

**Group reflection & discussion (15-20 mins)**

**1. Experience of the programme**

- How would you describe your experience of taking part in the CtW programme?
- What aspects of the CtW programme, were most helpful for you?
- What aspects of the CtW programme, were least helpful for you?
- Were there times that felt more difficult or frustrating for you?
- Do you feel you got the support and guidance you needed from the staff?

**2. Barriers and supports**

- What helped you move forward through the programme?
- What made things harder, and how did people overcome these challenges?

**3. Impact and outcomes**

- Looking back, what has changed for you since starting CtW?
- In what ways, has the CtW programme influenced your thinking or decisions about work in recent months?
- Which moments made the biggest difference in helping you move towards work?
- Have you found an employment?

**4. Collective meaning-making**

- If you were to describe the overall journey of the group, what words or images would you use?
- If someone new was joining CtW tomorrow, what advice would you give them about the journey ahead?

**5. Closing reflection**

- What is one thing you would like CtW to keep doing, and one thing you would like to see change?

**End of the workshop (5-10 mins)**

Summarise key insights.

Invite final reflections.

Thank participants
